# Abnormal Ca^2+^ homeostasis, atrial arrhythmogenesis, and sinus node dysfunction in murine hearts modeling *RyR2* modification

**DOI:** 10.3389/fphys.2013.00150

**Published:** 2013-06-25

**Authors:** Yanmin Zhang, Gareth D. K. Matthews, Ming Lei, Christopher L.-H. Huang

**Affiliations:** ^1^Department of Paediatrics, Institute of Shaanxi Province Children's Cardiovascular Diseases, The Shaanxi Provincial People's Hospital of Xi'an Jiaotong UniversityXi'an, PR of China; ^2^Faculty of Medicine and Human Sciences, Institute of Cardiovascular Sciences, University of ManchesterManchester, UK; ^3^Physiological Laboratory, Faculty of Biology, University of CambridgeCambridge, UK; ^4^Department of Biochemistry, University of CambridgeCambridge, UK

**Keywords:** *RyR2*, mutation, sinus node dysfunction, atrial arrhythmias, mouse models

## Abstract

Ryanodine receptor type 2 (*RyR2*) mutations are implicated in catecholaminergic polymorphic ventricular tachycardia (CPVT) thought to result from altered myocyte Ca^2+^ homeostasis reflecting inappropriate “leakiness” of RyR2-Ca^2+^ release channels arising from increases in their basal activity, alterations in their phosphorylation, or defective interactions with other molecules or ions. The latter include calstabin, calsequestrin-2, Mg^2+^, and extraluminal or intraluminal Ca^2+^. Recent clinical studies additionally associate *RyR2* abnormalities with atrial arrhythmias including atrial tachycardia (AT), fibrillation (AF), and standstill, and sinus node dysfunction (SND). Some *RyR2* mutations associated with CPVT in mouse models also show such arrhythmias that similarly correlate with altered Ca^2+^ homeostasis. Some examples show evidence for increased Ca^2+^/calmodulin-dependent protein kinase II (CaMKII) phosphorylation of RyR2. A homozygotic *RyR2-P2328S* variant demonstrates potential arrhythmic substrate resulting from reduced conduction velocity (CV) in addition to delayed afterdepolarizations (DADs) and ectopic action potential (AP) firing. Finally, one model with an increased RyR2 activity in the sino-atrial node (SAN) shows decreased automaticity in the presence of Ca^2+^-dependent decreases in *I*_Ca, L_ and diastolic sarcoplasmic reticular (SR) Ca^2+^ depletion.

## Introduction

Catecholaminergic polymorphic ventricular tachycardia (CPVT) is one of the most malignant cardiac channelopathies. It is characterized by episodic, life-threatening, arrhythmias provoked by stress and emotion in individuals with otherwise structurally normal hearts (Swan et al., 1999; Priori et al., 2001; Priori and Chen, 2011). Two genes have been implicated in CPVT. One, transmitted as an autosomal dominant trait, is caused by mutations in the ryanodine receptor type 2 (*RyR2*) gene (Laitinen et al., 2001; Priori et al., 2001). It accounts for 50–55% of the CPVT cases attributable to genetic abnormalities (Priori et al., 2002). The other, less common, recessive variant results from mutations in the cardiac-specific isoform of calsequestrin type 2 gene (*CASQ2*) (Lahat et al., 2001). There are currently more than 150 known pathological allelic variants involving *RyR2* that are associated with CPVT (Priori and Chen, 2011). Ventricular arrhythmia has been attributed in such cases to altered myocyte Ca^2+^ homeostasis. This results in an inappropriate “leakiness” of RyR2-Ca^2+^ release channels owing to increases in their basal activity, altered phosphorylation status or defective interactions with other molecules or ions, including calstabin (FKBP12.6) (Lehnart et al., 2004b), CASQ2, or Mg^2+^, or abnormal activation by extra- or intraluminal Ca^2+^ (Wehrens et al., 2004). This in turn results in spontaneous Ca^2+^ waves of Ca^2+^-induced Ca^2+^ release (CICR) in turn producing the delayed afterdepolarisation (DADs) thought to be the major cause of RyR2-associated CPVT (George et al., 2007; Mohamed et al., 2007; Priori and Chen, 2011). Seven *RyR2* mutations are additionally associated with atrial arrhythmic disorders that include atrial tachycardia (AT), fibrillation (AF), and standstill as well as sinus node dysfunction (SND) (Laitinen et al., 2001; Bhuiyan et al., 2007; Sumitomo et al., 2007; Beery et al., 2009; Marjamaa et al., 2009; Kazemian et al., 2011). RyR mutations can thus affect function in cardiac regions including sino-atrial node (SAN), atria, and atrioventricular (AV) node in addition to ventricular myocardium. This review surveys atrial arrhythmias and SNDs related to *RyR2* mutations as revealed by investigations in genetically modified murine cardiac models.

## Features of Ca2+ homeostasis in sino-atrial and atrial cells have implications for rhythm abnormalities

Atrial myocytes show differences from ventricular cells in their Ca^2+^ homeostasis in some important respects, with implications for the physiological consequences of alterations in their RyR2 function. Thus, atrial myocytes of small mammals lack extensive T-tubules (Mackenzie et al., 2001, 2004; Brette and Orchard, 2003). These are replaced by prominent, transversely-orientated, sarcoplasmic reticular (SR), Z-tubular, elements. Junctional RyR2–L-type Ca^2+^ channel (LTCC) clusters are confined to the cell peripheries in contrast to their dense distribution throughout the more extensive ventricular tubular system (Mackenzie et al., 2001). However, an abundant corbular SR contains non-junctional RyR2s (Jorgensen et al., 1993). Atrial activation is thus likely initiated by CICR following voltage-sensitive extracellular Ca^2+^ entry at the peripheral T-SR junctional elements. This initiates a centripetal propagation of this CICR process thereby increasing open probabilities in the corbular RyR2-Ca^2+^ release channels in the cell interior (Bootman et al., 2001; Mackenzie et al., 2001, 2004; Blatter et al., 2003; Sheehan and Blatter, 2003).

The detailed mechanisms leading to AF are poorly understood. Nevertheless, increasing evidence implicates alterations in intracellular Ca^2+^ signaling in its associated focal firing, substrate evolution and remodeling processes (Hove-Madsen et al., 2004; Vest et al., 2005; Dobrev and Nattel, 2008; Liang et al., 2008; Qi et al., 2008; Chelu et al., 2009; Dobrev, 2010). Acute atrial arrhythmogenesis in intact hearts in turn is related to diastolic Ca^2+^ events in atrial myocytes. These depend upon finite SR Ca^2+^ stores and diastolic CICR processes both of which ultimately depend upon extracellular Ca^2+^ entry. Thus, ectopic activity results from afterdepolarisation and triggered activity, whether arising from increased action potential duration (APD) resulting in early afterdepolarisation (EAD) or SR Ca^2+^ release resulting in DAD phenomena, and may trigger re-entrant activity or drive atrial rhythm at rapid rate resulting in fibrillatory conduction. However, reentry requires a vulnerable substrate, likely determined by the balance of conduction velocity (CV) and refractory period (Comtois et al., 2005; Dobrev and Nattel, 2008; Nattel et al., 2008).

Atrial myocytes from chronic AF patients similarly show increased frequencies of pro-arrhythmic spontaneous Ca^2+^ release events (Hove-Madsen et al., 2004; Vest et al., 2005). These took place despite reduced L-type voltage-dependent Ca^2+^ currents (Bosch et al., 1999; Van Wagoner et al., 1999; Workman et al., 2001; Christ et al., 2004) and have accordingly been attributed to increased RyR2-mediated Ca^2+^ release activity (De Bakker et al., 2002; Vest et al., 2005). Thus, RyR2-single-channel open probabilities were increased in canine hearts with persistent AF. AF is also associated with increased protein kinase A (PKA)-mediated RyR2 phosphorylation at S2808 and decreased calstabin binding to RyR2 (Vest et al., 2005). Such changes would be expected to cause a failure of RyR2 channel closure resulting in a potentially arrhythmogenic Ca^2+^ leak from the SR (Wehrens et al., 2004; Balasubramaniam et al., 2005; Chelu and Wehrens, 2007). However, the extent to which such a mechanism might be directly applicable to AF remains under discussion (Venetucci et al., 2007; Eckstein et al., 2008).

These general principles are likely to be applicable to atria in murine hearts, which have previously also provided useful models for studies of ventricular arrhythmic phenomena. Introduction of caffeine transiently induced diastolic Ca^2+^ waves in regularly stimulated atrial myocytes. This could result either from RyR2 sensitization to cytosolic Ca^2+^ or increased intracellular cAMP levels resulting from phosphodiesterase inhibition (Daly, 2007). However, this effect was inhibited by prior inhibition of either Ca^2+^-ATPase activity by cyclopiazonic acid or of extracellular Ca^2+^ entry by nifedipine. These latter findings in turn directly correlated with the induction of atrial arrhythmic tendency or its inhibition by the same agents in intact hearts (Zhang et al., 2009, 2011).

Possible roles for RyR2-Ca^2+^ release channels in normal SAN pacemaker activity remain under discussion (Mangoni and Nargeot, 2008; Lakatta and Difrancesco, 2009). The SAN contains both RyR2 and RyR3 as well as SR Ca^2+^ stores (Masumiya et al., 2003). Release of intracellularly stored Ca^2+^ tends to activate depolarizing inward Na^+^/Ca^2+^ exchange current (Rubenstein and Lipsius, 1989; Ju and Allen, 1998; Terrar and Rigg, 2000; Bogdanov et al., 2001; Lakatta et al., 2010). Should this occur at the end of diastolic depolarization it could potentially facilitate action potential (AP) firing. Such findings have given rise to suggestions for a “Ca^2+^ clock” (Bogdanov et al., 2001; Vinogradova et al., 2004). Certainly, SAN cells show high basal levels of cAMP that could increase PKA-dependent RyR2 phosphorylation modulating its release of Ca^2+^ and thereby potentially influencing SAN pacemaker function (Vinogradova et al., 2006). Ryanodine-mediated RyR2 block indeed reduces SAN beating frequency (Rubenstein and Lipsius, 1989; Ju and Allen, 1998; Bogdanov et al., 2001). However, others have suggested that such SR Ca^2+^ release is not a predominant factor in normal SAN pacemaker activity (Honjo et al., 2003; Mangoni and Nargeot, 2008; Lakatta and Difrancesco, 2009; Himeno et al., 2011).

## RyR2 mutations are associated with clinical atrial arrhythmic and SND phenotypes

A number of *RyR2* mutations associated with CPVT are also associated with atrial arrhythmias and SND (Table 1). However, there have been no reported cases of lone AF or SAN dysfunction in the absence of CPVT. Large deletions encompassing exon 3 in RyR2 NH_2_-terminal regions are relatively frequent. This may reflecting their containing *Alu* repeats predisposing to deletions resulting from large *Alu* repeat–mediated genomic rearrangements resulting from polymerase slippage (Gu et al., 2008). *Alu* sequences occur in intron 2, 190 bp upstream from exon 3 and also 536 bp downstream in intron 3. They are related to the 1.1 kb deletion containing part of intron 2, exon 3, and part of intron 3, with a *Alu-Alu* recombination in which exon 2 is recombined with exon 4 and exon 3 is completely deleted (Bhuiyan et al., 2007; Marjamaa et al., 2009; Medeiros-Domingo et al., 2009). However, crystal structure studies revealed that exon 3 deletion causes a structural rescue whereby a flexible loop inserts itself into the β trefoil domain. This increases the thermal stability of the RyR2 channel. As a result the mutation is neither lethal nor confers loss of function (Lobo et al., 2011). In the HEK293 expression system, it results in a marked reduction in the luminal Ca^2+^ threshold at which Ca^2+^ release terminates thereby increasing fractional Ca^2+^ release (Tang et al., 2012). The RyR2 NH_2_-terminal region may thus be an important determinant for termination of Ca^2+^ release, abnormalities in which are common in RyR2-associated cardiomyopathies (Gu et al., 2008; Tang et al., 2012).

**Table 1:** **Summary of RyR2 mutations associated with atrial and/or sinoatrial node dysfunction**.

**Nucleotide**	**Amino acid change**	**Mutation**	**Location**	**Exon**	**Phenotype**	**References**
1.1 kb deletion	Exon 3 deletion	Deletion	Amino terminal domain	3	CPVT, SND, AV nodal block, AF, and atrial standstill, DCM	Bhuiyan et al., 2007; Marjamaa et al., 2009; Medeiros-Domingo et al., 2009
C6982T	P2328S	Missense	FKBP binding domain	46	CPVT, AT, Normal heart structure	Laitinen et al., 2001
-No information	G3946A	Missense	Cytosol	88	CPVT, AT, AF, Normal heart structure	Pizzale et al., 2008
T12056G	M4109R	Missense	Domain II	90	CPVT, AF, transient QT prolongation during AF, and postpacing, Normal heart structure	Nof et al., 2011
A12457C	S4153R	Missense	Domain II	90	CPVT, AF, Normal heart structure	Kazemian et al., 2011
-No information	W4645R	Missense	Transmembrane domain	96	CPVT, AF, Normal heart structure	Beery et al., 2009
-No information	A7420G	Missense	C-Terminal domain	105	CPVT, AF, Normal heart structure	Sumitomo et al., 2007

A large exon 3 *deletion* has been reported in a number of unrelated families. It has been associated with a broad range of atrial, ventricular and SND phenotypes. Thus, several unrelated families showed a large deletion encompassing part of intron 2, exon 3, and part of intron 3, expected to result in an in-frame deletion of 35 amino acids (p.Asn57-Gly91) (NM-001035) (Bhuiyan et al., 2007; Marjamaa et al., 2009; Medeiros-Domingo et al., 2009). This deletion was associated with a spectrum of phenotypes including SND, AF, atrioventricular block (AVB), decreased left ventricular function, increased trabeculation, CPVT, and dilated cardiomyopathy (DCM). Two further families showing CPVT with large deletions in the region did not show the association with DCM (Marjamaa et al., 2009). Mutation carriers in one of these families showed CPVT, AF, sinus bradycardia, and AV conduction abnormalities without structural cardiovascular abnormalities. Patients from the other family showed increased left ventricular trabeculation suggestive of non-compaction cardiomyopathy (Marjamaa et al., 2009). An additional report of a large large 3.6 Kb exon 3 deletion was not accompanied by a description of the corresponding patient phenotype (Medeiros-Domingo et al., 2009).

Some single CPVT-associated *RyR2* mutations are also associated with atrial arrhythmias. Two such families, including one with *RyR-P2328S*, contained 14 CPVT patients (Swan et al., 1999; Laitinen et al., 2001). All carriers showed polymorphic ventricular tachycardia (PVT) and/or ventricular fibrillation (VF) of which three patients also showed non-sustained AT. Three further case reports described a further three *RyR2* mutations all of which showed AT and/or AF in addition to CPVT without abnormalities in cardiac structure (Sumitomo et al., 2007; Pizzale et al., 2008; Kazemian et al., 2011). Thus, a patient with the *RyR2-G3946A* variant who had an implantable cardioverter defibrillator (ICD) for CPVT showed AT/AF both during exercise and on ambulatory electrocardiographic (ECG) recording (Pizzale et al., 2008). The *RyR2-S4153R* variant was identified in a women presenting with a cardiac arrest due to VF; she showed AF on 12-lead ECG immediately following emergency defibrillation. Over a 12 month follow-up period, in addition to experiencing three ICD shocks related to appropriately detected PVTs, she showed several exercise-induced self-terminating episodes of AF with rapid ventricular responses (Kazemian et al., 2011). Finally, a *RyR2-A7420G* variant was identified in a patient with both clinical and induced AF as well as frequent episodes of paroxysmal AF during walking or with mental stress despite normal sinus node recovery times (SNRT) and cardiac structure (Sumitomo et al., 2007).

In a further clinical situation, a preceding rapid AF could initiate CPVT, after a marked transient QT prolongation following a long RR interval that in turn followed a sequence of short R-R beats. This was associated with two heterozygous nucleotide substitutions, *RyR2-M4109R* and *RyR2-I406T*. All the *RyR2-M4109R* but none of the *RyR2-I606T* carriers showed prominent postpacing QT prolongation during electrophysiological study, although both showed normal QT intervals during regular rhythm (Nof et al., 2011). Finally, VT and AT could be induced by isoproterenol infusion, an effect blocked by propranolol administration, in a mother and son both having a *RyR2-W4645R* mutation and clinical diagnoses of PVT and AT in an absence of detectable significant underlying cardiac disease (Beery et al., 2009).

## Atrial arrhythmic and Ca2+ homeostatic abnormalities in mouse models harboring RyR2 mutations

Increased diastolic Ca^2+^ release has been implicated in generation of ventricular arrhythmias in genetically modified mouse models harboring CPVT-related *RyR2* mutations (Cerrone et al., 2005; Kannankeril et al., 2006; Goddard et al., 2008; Lehnart et al., 2008; Suetomi et al., 2011). Recently, a few mouse models have similarly shown atrial arrhythmias and SND and similarly demonstrated evidence for diastolic SR Ca^2+^ release through “leaky” RyR2s (Chelu et al., 2009; Suetomi et al., 2011; Zhang et al., 2011; Neco et al., 2012; Shan et al., 2012).

*RyR2-R176Q*/+ mice (Kannankeril et al., 2006) did not show spontaneous AF but nevertheless showed increased vulnerabilities to AF during rapid atrial pacing when compared to wild-type (WT). This was associated with increased Ca^2+^/calmodulin-dependent protein kinase II (CaMKII) phosphorylation of RyR2. Conversely, pharmacological inhibition of CaMKII prevented this AF inducibility. So did genetic inhibition of CaMKII-mediated RyR2 phosphorylation in *RyR2-S2814A* mice studied in a vagotonic AF model. Atrial biopsies from mice with atrial enlargement and spontaneous AF, goats with lone AF, and patients with chronic AF similarly showed increased CaMKII phosphorylation of RyR2. These findings implicate a RyR2-dependent Ca^2+^ release resulting from increased CaMKII activity in susceptibility to AF (Chelu et al., 2009).

**Figure 1:**
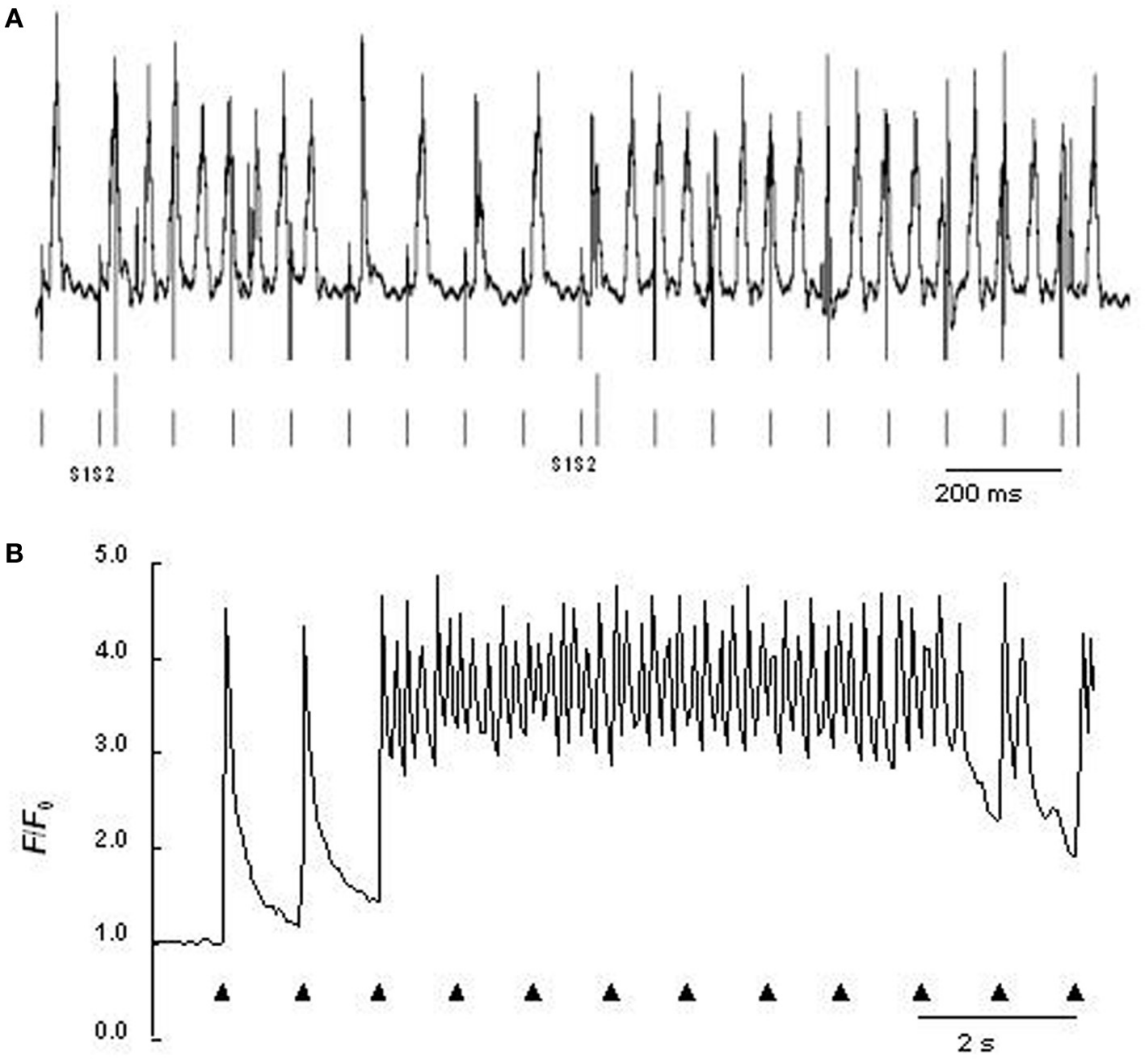
**(A)** Atrial monophasic action potential recording of arrhythmic events provoked by programmed electrical stimulation involving extrasystolic S2 stimulation (long vertical bars beneath trace) following series of pacing S1 stimuli (short vertical bars beneath trace) from isolated perfused homozygotic *RyR2-P2328S* heart. **(B)** Ca^2+^ transients from regularly stimulated fluo-3-loaded *RyR2-P2328S* atrial myocytes under confocal microscopy, both following introduction of isoproterenol [From Figures 5D, 6F of Zhang et al. (2011)].

The *RyR2-P2328S* mutation has been clinically related to atrial in addition to ventricular arrhythmic phenotypes (Swan et al., 1999; Laitinen et al., 2001). Heterozygotic and homozygotic *RyR2-P2328S* mouse atria both showed acute arrhythmogenic properties in the absence of structural abnormalities with a severity related to gene dose, consistent with previous results in expression systems (Lehnart et al., 2004a). Intact anaesthetized hetero- or homozygotic *RyR2-P2328S* showed normal electrocardiographic parameters both before and after isoproterenol addition apart from increased heart rates. Nevertheless, bipolar electrogram and monophasic AP recordings from either regular or programmed stimulation demonstrated higher atrial arrhythmogenic incidences in isolated perfused homozygotic but not heterozygotic *RyR2-P2328S* relative to WT. Isoproterenol increased these incidences in all three groups despite unchanged AP durations at 90% recovery (APD_90_) and atrial effective refractory periods (AERP) (Figure 1A). These findings correlated with occurrences of diastolic Ca^2+^ release phenomena in regularly stimulated, isolated, fluo-3-loaded *RyR2^S/S^*, but not *RyR2^+/S^* or WT, atrial myocytes, whose frequency was increased by isoproterenol (Figure 1B) (Zhang et al., 2011). Finally, microelectrode recordings in isolated perfused homozygotic *RyR2-P2328S* atria showed DADs and ectopic APs not found in WT that could potentially result from such Ca^2+^ release phenomena (King et al., 2013). These also further implicated atrial arrhythmic substrate resulting from reduced CV in agreement with similar results bearing on ventricular conduction (Zhang et al., 2013). This was reflected in their increased interatrial conduction delays, reduced epicardial CVs and reduced maximum rates of AP depolarization [(d*V*/d*t*)_max_], despite similar effective refractory periods, AP durations and AP amplitudes.

Finally, AF could be induced by an intra-esophageal burst pacing protocol in mice harboring the *RyR2-R2474S*+/−, *RyR2-N2386I*+/−, and *RyR2-L433P*+/− mutations known to be associated with human CPVT. Their isolated atrial myocytes showed significant diastolic SR Ca^2+^ leaks. Atrial RyR2s from *RyR2-R2474S*+/− mice were oxidized and their RyR2 complexes depleted of the calstabin2 unit known to stabilize the RyR2 closed state. The Rycal agent S107, known to stabilize RyR2-calstabin2 interactions and inhibit the oxidation/phosphorylation-induced dissociation of calstabin2 from the channel, rescued the resulting increase in Ca^2+^ release, and prevented burst pacing–induced AF *in vivo*. It did not do so in calstabin2-deficient mice, implicating calstabin2 in these arrhythmic properties (Shan et al., 2012).

## Paradoxical sinus node disorder in mouse models harboring RyR2 mutations

In contrast to experimental evidence implicating a Ca^2+^ clock involving RyR2 in pacemaker activity (see section on Features of Ca^2+^ homeostasis in sino-atrial and atrial cells have implications for rhythm abnormalities), CPVT is associated with high incidences of SAN dysrhythmia in the form of *reduced* SAN automaticity resulting in basal bradycardia, sinus pauses, and impaired chronotropic responses to β-adrenergic stimulation (Leenhardt et al., 1995; Sumitomo et al., 2003; Postma et al., 2005; Katz et al., 2009). Although they do not show a basal bradycardia, *RyR2-R4496C* mice studied using *in vivo* telemetric recordings similarly showed sinus pauses overcome by atrial and junctional escapes triggered by catecholamines, and impaired SAN automaticity following isoproterenol injection. These features paralleled post-exercise findings in CPVT patients (Neco et al., 2012). Measurements of spontaneous [Ca^2+^]_i_ transients reflecting pacemaker activity in SAN cells by confocal microscopy correspondingly demonstrated slower pacemaker activity and impaired chronotropic responses to β-adrenergic stimulation, and sinus pauses in 75% of the cases. These changes were associated with 50% reductions in L-type Ca^2+^ current (*I*_Ca, L_) density in whole-cell patch-clamped *RyR2-R4496C* SAN cells. Isoproterenol dramatically increased the frequency of Ca^2+^ sparks and Ca^2+^ waves by ~5 and ~10-fold (Neco et al., 2012).

## Conclusion

Enhanced RyR2 activity associated with *RyR2* mutations can be associated with atrial arrhythmic tendency and SND. This is potentially attributable to increased RyR2-mediated release of intracellularly stored, SR, Ca^2+^ release and altered interactions with CaMKII and calstabin2. Increased RyR2 activity in the SAN leads to an unanticipated decrease in its automaticity accompanied by a Ca^2+^-dependent decrease of *I*_Ca, L_ and diastolic SR Ca^2+^ depletion.

## Funding source

This work was supported by the Medical Research Council, Wellcome Trust, British Heart Foundation, the Biotechnology and Biological Sciences Research Council, and Natural Science Foundation of China (30371571, 30830051, and 30672209).

### Conflict of interest statement

The authors declare that the research was conducted in the absence of any commercial or financial relationships that could be construed as a potential conflict of interest.
